# Optimisation of an Aglycone-Enhanced Celery Extract with Germinated Soy Supplementation Using Response Surface Methodology

**DOI:** 10.3390/foods10102505

**Published:** 2021-10-19

**Authors:** Hazel Lau, Hui Min Koh, Hiranya Dayal, Yi Ren, Sam Fong Yau Li

**Affiliations:** 1 Department of Chemistry, National University of Singapore, 3 Science Drive 3, Singapore 117543, Singapore; hazel_lau@u.nus.edu (H.L.); e0506465@u.nus.edu (H.M.K.); chmhda@nus.edu.sg (H.D.); 2 Institute of Materials Research and Engineering, Agency for Science, Technology and Research (A∗STAR), 2 Fusionopolis Way, Innovis, #08-03, Singapore 138634, Singapore; reny@imre.a-star.edu.sg; 3 NUS Environmental Research Institute (NERI), #02-01, T-Lab Building (TL), 5A Engineering Drive 1, Singapore 117411, Singapore

**Keywords:** celery, germinated soy, response surface methodology, aglycone, apigenin

## Abstract

In this study, the extraction conditions of bioactive aglycones from a celery extract supplemented with germinated soy were optimised by a response surface methodology. For subsequent enzymatic hydrolysis to enhance the apigenin content, increased production of its precursor apigetrin was firstly achieved through acidic extraction at optimal conditions, involving water at pH 1, at 75 °C for 2 h. Subsequently, a central composite design was conducted to analyse the pH (3–11) and temperature (25–35 °C) effects on the aglycone levels (apigenin, daidzein and genistein). The optimal extraction conditions were pH 7.02 and 29.99 °C, which resulted in a 40-fold increase in apigenin. The novel and cost-effective application of germinated soy β-glucosidase for the conversion of aglycones in non-soy foods is demonstrated. The enhanced bioactivities of aglycones may suggest potential applications for similar formulations as functional food ingredients.

## 1. Introduction

Celery (*Apium graveolens* L. var. *dulce*) is a globally cultivated vegetable from the Mediterranean and Middle East known to be a rich source of nutrients and flavonoids [1]. The key flavonoid in celery has been identified as apigenin, which is present in aglycone and glycoside forms such as apiin (apigenin-7-apioglucoside) and apigetrin (apigenin-7-glucoside). Compared to its glycosides, apigenin has been reported to have more potent bioactivity in the form of stronger anti-inflammatory [2] and antioxidant [3] properties. Apigenin has also independently garnered interest due to its wide range of pharmacological benefits including anti-carcinogenic and anti-toxicant effects [4]. As such, various strategies to enhance the apigenin content in celery have been implemented, including increasing flavone synthase gene expression [5] and enzymatic hydrolysis by pectinase for increased accessibility to intracellular contents [6] and by β-glucosidase [7] and β-glucosidase-rich ingredients [8]. The celery stem, which constitutes the majority of the celery plant, is the more suitable target for the improvement of apigenin content, as apigenin accumulation is less prominent in celery stems compared to the leaves [9].

The use of germinated soybeans in food applications has become increasingly popular due to the activation of enzymes such as β-glucosidase during the germination process, leading to enhanced bioactive properties [10]. Specifically, glycosidic isoflavones (daidzin, genistin) are converted into aglycones such as daidzein and genistein by β-glucosidase activity in soy, and these aglycones are known to provide protective effects against chronic diseases such as diabetes, hypertension and atherosclerosis [11]. Clinical evidence for the increased bioavailability of isoflavones in the aglycone form [12,13] has prompted multiple studies on enhancing soy isoflavone conversion to the aglycone form via microbial and germinated soy β-glucosidase [14,15]. In particular, germinated soy β-glucosidase was found to enable isoflavone conversions comparable to microbial sources, and was noted to have the added benefit of supply stability afforded by an endogenous source [15]. These findings suggest that germinated soy may have great potential to facilitate aglycone conversion in other foods, such as celery, which have yet to be explored.

For the optimisation of aglycone conversion from celery and soy flavonoid glycosides, investigation into the effect of extraction parameters requires a systematic approach. There have been varying results reported by different authors regarding the optimal conditions for soy β-glucosidase activity and isoflavone extraction: Yoshiara et al. identified the optimal extraction of soy β-glucosidase at pH 5 and 30 °C, while soy isoflavone recovery was best at pH 7 and 35 °C [15]. Other authors reported the optimum conditions of pH 5 and 45 °C [16], as well as pH 6 and 30 °C [17], which indicates potential differences based on the soy used. Studies on β-glucosidase conversion of apigetrin to apigenin are lacking, which is the main focus of the present work. For efficient optimisation of the variables, the application of a response surface methodology (RSM) is ideal and has been previously applied to evaluate the extraction conditions in multiple food samples ranging from turnip slices [18] to vinegar [19]. This may be attributed to its benefits such as simultaneous parameter optimisation, which accounts for interactive effects through statistical modelling and effective reduction of the number of measurements required via experimental designs such as central composite design (CCD) [20].

The present study aimed to optimise the extraction of aglycones from a celery extract supplemented with germinated soy. Extraction of the glycoside precursor in celery, apigetrin, was conducted, followed by systematic optimisation of the parameters using RSM to facilitate the conversion of aglycones present in celery and soy (apigenin, daidzein, genistein). The novel and cost-effective application of germinated soy β-glucosidase for the conversion of aglycones in non-soy foods is demonstrated, and the enhanced bioactivities of aglycones may suggest potential applications of similar formulations as functional food ingredients.

## 2. Materials and Methods

### 2.1. Chemicals

Formic acid and FMOC-glycine were purchased from Sigma Aldrich (St. Louis, MO, USA), HPLC-grade ethanol and 37.5% HCl were purchased from Merck (Kenilworth, NJ, USA), 30% NaOH was purchased from VWR (Radnor, PA, USA) and LC-MS grade methanol was obtained from Thermo Fisher Scientific (Waltham, MA, USA).

### 2.2. Preparation of Plant Material

For the preparation of celery stem powder, whole celeries (individually wrapped and labelled) from China were purchased from local supermarkets in Singapore, and the celery stems were obtained and washed with deionised water before freeze-drying in a Labconco FreeZone freeze-dryer (Kansas City, MO, USA). The freeze-dried samples were ground and stored in light-protected centrifuge tubes at −80 °C prior to extraction. Published reports of β-glucosidase activity in freeze-dried soy [15,21] and the successful isolation of β-glucosidase from soy stored at −80 °C [22] indicated that freeze-drying would not eliminate β-glucosidase activity, and it was, therefore, selected as a suitable method of drying for this experiment.

For the preparation of germinated soy powder, the protocol was partially adapted from Yang et al. [23]: Canadian soy bean seeds (NTUC Supermarket, Bukit Panjang, Singapore) purchased from a local supermarket were washed and cleaned with 0.5% sodium hypochlorite before sonicating at room temperature (25 °C) for 30 min in an ultrasonicator (Elmasonic S 60H, 37 kHz, Elma Schmidbauer GmbH, Singen (Hohentwiel), Germany). Subsequently, the seeds were placed on wet cotton cloth in the dark for germination at room temperature. The seeds were sprayed with deionised water every 6 h to maintain an adequate hydration level. After germination (48 h), the seeds were freeze-dried in a Labconco FreeZone freeze-dryer (Kansas City, MO, USA). The freeze-dried samples were ground and stored in light-protected centrifuge tubes at −80 °C prior to extraction.

### 2.3. Extraction Procedure

For apigetrin extraction from celery stems, 0.2 g of sample was added to 8 mL glass vials, followed by 2 mL of extraction solvent containing FMOC-glycine as an internal standard (1 µg/mL). The effect of different pH levels (pH 1–3) in water was evaluated, followed by the effect of different ethanol contents in water (0/100, 25/75, 50/50, 75/25, 100/0; *v/v*) at pH 1. Subsequently, extraction was carried out in an ultrasonicator (Elmasonic S 60H, 37 kHz, Elma Schmidbauer GmbH, Singen (Hohentwiel), Germany) at different temperatures (35 °C, 55 °C, 75 °C) for different time intervals (1 h, 2 h, 3 h). After extraction, the vials were cooled to room temperature and centrifuged (9000 rpm for 10 min and transferred to 1.5 mL microcentrifuge tubes, followed by 14,800 rpm for 10 min) in 5804R and 5430R benchtop centrifuges (Eppendorf, Hamburg, Germany), respectively, before filtering with 0.2 µM PTFE filters (Sartorius, Göttingen, Germany). In this part of the experiment, the extraction variables were independently optimised and carried out using the optimal conditions obtained beforehand. Our preliminary experiments indicated that for the extraction variables involved in apigetrin extraction, most of the responses were optimal at the feasible experimental limits, so a multivariate experimental design was not conducted.

For the extraction of apigenin and soy aglycones, the addition of germinated soy powder served to enhance aglycone levels in the apigetrin extract. The current amount of soy powder used was the highest feasible mass to be added with complete immersion in the celery extract in preliminary trials. As there was more than one substrate present in the experiment (apigetrin, genistin and daidzin), it was not possible to optimise the ratio as envisioned for a traditional enzyme experiment. The priority was thus to maximise the amount of soy, to enable higher quantities of the soy isoflavones and β-glucosidase to be present for the production of the aglycone-rich extract. In this section, 0.04 g of germinated soy powder was added to 0.4 mL of apigetrin extract (pH adjusted as per experimental plan, Table 1). The samples were incubated for 2 h in a water bath at set temperatures (Table 1). After extraction, the vials were cooled to room temperature and centrifuged (14,800 rpm for 15 min) before filtering with 0.2 µM PTFE filters (Sartorius, Göttingen, Germany).

### 2.4. Experimental Design and Model Verification

Response surface methodology (RSM) with central composite design (CCD) was employed to optimise two parameters, pH (X_1_) and incubation temperature (X_2_), for the extraction of apigenin and soy aglycones. Twelve experiments were performed in a randomised order and comprised a complete 2^2^-factorial design as cubic points, with four axial points at a distance of α = 1.414 from the design centre and four centre points (Table 1). The optimal values of the parameters were obtained by solving the regression equations and by analysing the response surface and contour plots of the fitted RSM equations, and the predicted values were compared with the experimental value to determine the model validity.

### 2.5. LC-MS/MS

Samples were analysed on a Dionex Ultimate 3000 HPLC system (Thermo Fisher Scientific, Waltham, MA, USA) coupled to a QTRAP 5500 system (AB Sciex, Framingham, MA, USA). Compounds were ionised with polarity switching using electrospray ionisation with the following source conditions: nebuliser gas = 70 psi, heater gas = 55 psi, curtain gas = 40 psi, heater temperature = 650 °C, ionspray voltage = 5400/−4500 V. Multiple reaction monitoring (MRM) was employed for quantitation with an accumulation time of 30 ms per ion pair, and the precursor-to-product ion pair, declustering potential (DP) and collision energy (CE) for each analyte are described in Table S1. The chromatograms were integrated using Analyst 1.5.1 (AB Sciex, Framingham, MA, USA), and the peak areas obtained were used for quantification based on the external calibration of standard solutions, with the analytical figures of merit described in Table S1.

Chromatographic separation on the LC-QTrap was achieved with 10 µL injections on a Zorbax Eclipse C18 column (100 mm × 2.1 mm, 3.5 µM) (Agilent Technologies Inc., Santa Clara, CA USA) at a flow rate of 0.5 mL/min at 40 °C, and mobile phases A and B were 0.1% formic acid in water and 0.1% formic acid in methanol, respectively. The following gradient was used: 10% B for 2 min, increased to 40% B at 2.5 min, ramped up to 85% B at 7.5 min, followed by another increase to 95% B at 7.6 min, held for 1.9 min, then dropped to 10% B over 0.1 min and held for 2.4 min.

### 2.6. Data Analysis

Statistical analyses were performed with R version 4.0.2 [24] using a public package. The CCD experiment design and response surface plots were generated using the package ‘rsm’ [25] in RStudio (Version 1.2.5019, R. RStudio, Inc., Boston, MA, USA).

## 3. Results and Discussion

### 3.1. Extraction Optimisation of Apigetrin

**Figure 1:**
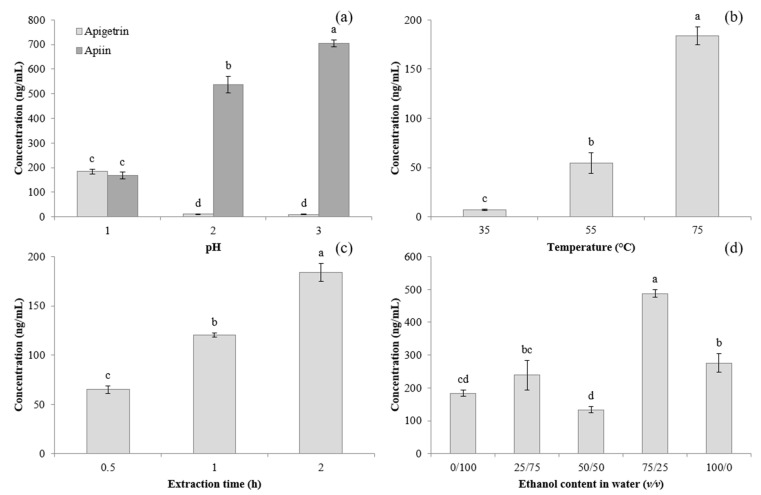
Extraction optimisation of apigetrin: (**a**) effect of pH in water at 75 °C for 2 h; (**b**) effect of temperature in water at pH 1 for 2 h; (**c**) effect of extraction time in water at pH 1 and 75 °C; (**d**) effect of ethanol content in water at pH 1 and 75 °C for 2 h. Different letters indicate a significant difference (*p* < 0.05).

For enhanced apigenin content in subsequent enzymatic hydrolysis, increased production of its precursor apigetrin was firstly achieved through acidic extraction, which included simultaneous extraction from the celery matrix and hydrolysis of apiin to apigetrin. This process was found to be affected by a range of extraction parameters such as pH, temperature, time and ethanol content, as can be seen in Figure 1. From Figure 1a, it can be observed that the pH had a significant effect on the extraction of apigetrin, with pH 1 resulting in a 20-fold increased recovery compared to pH 2 and pH 3, which corresponded with the reduction in apiin concentration due to acid hydrolysis. This indicates the effectiveness of highly acidic conditions in the hydrolysis of the apiose group from apiin, which was previously investigated by Hostetler et al. at a higher pH range (3–7) and higher temperature (100 °C), with substantial apiin degradation occurring at pH 3 in contrast to pH 5 and pH 7 [8].

Increased extraction efficiencies may be expected with higher temperatures due to a higher analyte solubility and mass transfer rate, supported by thermal disruption of the celery matrix as well as lower viscosity and surface tensions of the extraction solvent, although longer extraction times may result in degradation [26]. However, apigetrin extraction appeared to be more efficient at a higher temperature (75 °C) and over a longer duration (2 h), as demonstrated in Figure 1b,c. A longer extraction time of 3 h was tested in preliminary trials, which resulted in a decreased hourly rate of apigetrin enhancement compared to a shorter extraction time of 2 h. As such, it was determined to be of little benefit to extend the extraction time for an extra hour, since the main objective of the paper was to focus on apigenin conversion.

Apigetrin had previously demonstrated notable thermal stability at 100 °C with less than 10% degradation after 5 h [8], and extraction studies on apigetrin in other matrices such as chamomile and marjoram generally reported increasing extraction efficiencies with temperature and time [27,28]. An exception occurred in one study on chrysanthemum, which found that extraction was optimal at 50 °C [29], suggesting that in addition to possible matrix effects, the higher temperature and time required may generally be more closely related to apiin degradation than the extraction process, since apiin levels in chrysanthemum appear to be low [30].

While acidified organic solvents are conventionally used in the extraction of phenolic compounds [26], apigetrin contains a glucoside group that increases its solubility in water and enables aqueous extractions [29]. Contrasting findings have been reported for apigetrin extraction, which indicates the strong influence of the matrix components: 30% ethanol appeared to improve the extraction efficiency more so than 50% or 70% in sage [31], while 50% ethanol and above was superior in *Hieracium pilosella* [32]. While the experimentally determined optimum ethanol content in this work was 75/25 (*v/v*), as shown in Figure 1d, it was observed in preliminary trials that apigenin formation was suppressed despite reports of ethanol-tolerant β-glucosidases [33]. As such, aqueous apigetrin extracts were used in the subsequent optimisation with germinated soy supplementation, which were also considered an environmentally friendly alternative to the use of organic solvents.

### 3.2. Extraction Optimisation of Apigenin and Soy Aglycones

Non-enzymatic hydrolysis of apigetrin to apigenin was previously found to be optimal at a lower pH (pH 1.1) in chrysanthemum and facilitated by high temperatures (80 °C). However, the hydrolysis rate remained below 8% [29], indicating poor conversion efficiency to apigenin. As such, supplementation with germinated soy powder containing β-glucosidase, which has previously been found to enable high isoflavone conversions comparable to microbial sources, is introduced in this work as a novel approach to obtain higher levels of apigenin. The presence of bioactive aglycones (daidzein, genistein) in germinated soy is also considered to contribute additional health benefits to the final germinated soy-supplemented celery extract.

The temperature and pH dependencies of β-glucosidases have been reviewed and found to differ greatly based on their origin. The optimal condition can range from pH 2.4 in *T. cylindrosporum* to pH 10 in *Chaetomium globosum*, and the optimal temperature can be as high as 105 °C for *Pyrococcus furiosus* [33]. For soy β-glucosidases, the reported ranges have been from pH 5 to 7 and from 30 to 45 °C [15,16,17]. As such, it is necessary to further confirm the optimal extraction parameters for the extraction of multiple aglycones, especially for the conversion of apigetrin to apigenin, which is not indigenous to soy.

#### 3.2.1. Validation of the Experimental Design

Table 1 shows the experimental conditions for the extraction of aglycones with germinated soy supplementation, giving the predicted and experimental values of apigenin, daidzein and genistein. The extractions and analytical runs were performed on the same day. Apigenin, daidzein and genistein ranged from 2.86–38.94 ng/mL, 2.38–8.91 µg/mL and 9.66–37.65 µg/mL, respectively, and the maximum observed concentration obtained for the aglycones occurred at the 12th run (pH 7, 30 °C). These findings are close to the reported values for other soy β-glucosidases: Yoshiara et al. identified that the optimal extraction of soy β-glucosidase occurred at pH 5 and 30 °C, while soy isoflavone recovery was best at pH 7 and 35 °C [15]. Other authors reported optimum conditions of pH 5 at 45 °C [16], as well as pH 6 at 30 °C [17]. It was also noted that a negative value was predicted on the 1st run for apigenin. The reason is that due to the curvature of the model, the prediction accuracy is best at the centre points where the optimal condition is defined instead of the model limits. Hence, levels of the compound close to zero may be reflected as a negative value. Although a forced positive model fit would remove the negative value, such an approach would negatively affect the model performance and hence was not adopted.

To explain the adequacy and significance of the second-order polynomial models, Table 2 presents statistical parameters obtained from a Fisher’s F-test of the models, including the individual variables of the fitted response models. *p*-values of the apigenin and daidzein models indicated overall model significance (*p* < 0.05), while genistein had a higher *p*-value of 0.115. The coefficients of determination (R^2^)—which were at 0.915, 0.788 and 0.706 for apigenin, daidzein and genistein, respectively—revealed the quality of the model fit. The high *p*-value (*p* > 0.05) and low adjusted R^2^ value of genistein (R^2^ < 0.6) highlight a lack of model adequacy for this aglycone. As the lack of fit *p*-values were all >0.05, there was no further evidence of model inadequacy. For the individual variables, it was clear that pH was the factor with a significant effect, in contrast to temperature, as the X_1_^2^ term was significant (*p* < 0.05) with a negative coefficient across all responses, suggesting that a clear pH optimum was discovered within the tested range.

#### 3.2.2. Analysis of the Response Surface

**Figure 2:**
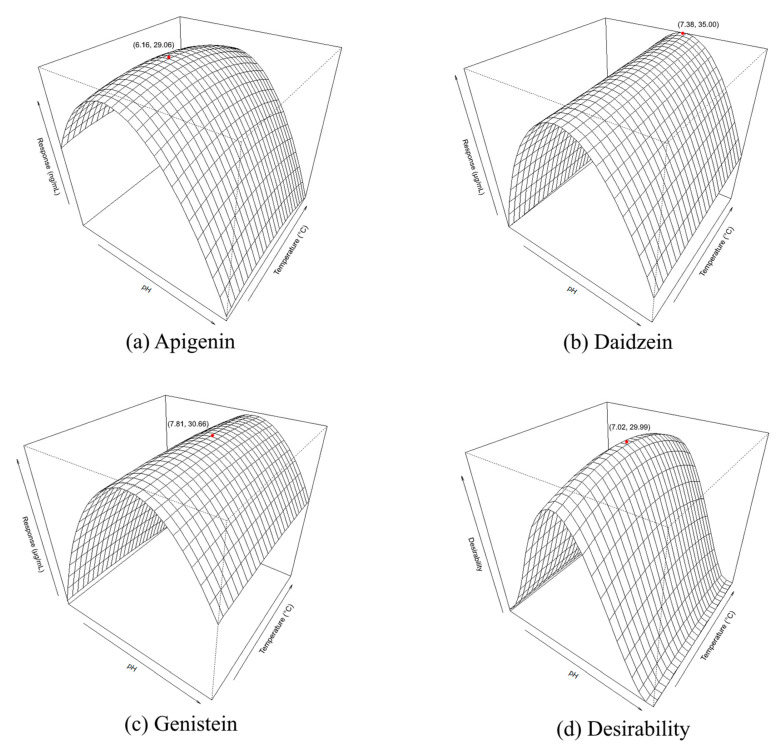
Response surface plots for the visualisation of interaction effects between pH and temperature (°C) on apigenin, daidzein, genistein and the desirability index for combined responses.

Figure 2 was generated using the RSM model for the visualisation of interaction effects between independent (pH and temperature) and dependent variables (apigenin, daidzein, genistein and desirability). Based on the figures, lower responses generally occurred at the limits of the tested range, while the highest responses appeared to be located around a similar pH region of the response surface plots, and were calculated to be (1) pH 6.16 at 29.06 °C for apigenin, (2) pH 7.38 at 35 °C for daidzein, (3) pH 7.81 at 30.66 °C for genistein and (4) pH 7.02 at 29.99 °C for the desirability (collective aglycone) response.

For all aglycones, pH has a major influence on the responses in agreement with the significant X_1_^2^ term in Table 2, which may be attributed to the reported optimal pH from pH 5 to 7 for soy β-glucosidase activity [15,16,17]. Furthermore, as apigenin, daidzein and genistein have all been reported to be less stable at a higher pHs [8,34], the pH stability of the aglycones may partially explain the sharp decrease in recovery at higher pH values. It was also noted that while apigenin is supposedly the most stable below pH 5 [8], the optimum value for recovery was, however, slightly higher at pH 6.16, suggesting that it was more critical to optimize the β-glucosidase activity in this experiment.

Compared to apigenin, the extractions of daidzein and genistein appeared to be largely unaffected by temperature. In view of the insignificant *p*-values (*p* > 0.05) of the temperature variables, as shown in Table 2, we concluded that the effect of temperature was insignificant within the current tested range. While higher temperatures have been reported to enhance extractions of daidzein and genistein from soy [35], initial screening indicated decreasing recoveries of apigenin above 35 °C. In addition, as the lowest feasible temperature was 25 °C due to equipment limitations, temperatures from 25 °C to 35 °C were investigated for aglycone optimisation. At the lower temperature range presented in this study, the temperature-independent aglycone conversion from daidzin and genistin by β-glucosidase was more likely to be the dominant pathway for the production of aglycones, accounting for these findings. Overall, it is suggested that β-glucosidase is largely responsible for the enhancement of apigenin in the soy-supplemented celery extract for the following reasons: (1) germinated soy has previously been ascertained to be rich in β-glucosidase, which facilitates soy aglycone conversion [15], and our optimal condition corresponded well to the reported pH and temperature ranges in the literature (pH 5–7, 30–45 °C) [15,16,17]; (2) degradation of apigetrin to apigenin at the tested conditions (pH 3–11, 25–35 °C) was expected to be insignificant based on stability observations from a previous publication [8]; (3) the bond broken is a strong glycosidic bond, which requires hydrolysis by a specific enzyme (β-glucosidase) or acid hydrolysis (which has been reported to occur at a rate of near 0% even after 8 h [29]).

#### 3.2.3. Optimisation Responses and Verification of the Model

Based on the data obtained, the extraction process was optimised for the collective response via a desirability function [36]. By solving the maximisation problem with desirability as the objective, the optimal conditions were determined to be pH 7.02 at 29.99 °C. The model prediction response was verified by comparing the observed and predicted value of the responses (Table 3), with insignificant deviation (*p* > 0.05), verifying the prediction accuracy.

#### 3.2.4. Quantitation of Bioactive Compounds by LC-MS/MS Analysis

Key bioactive compounds in celery and soy were quantitated in the apigetrin-optimised celery extracts and germinated soy-supplemented extracts (Table 4). A 40-fold increase of apigenin was obtained after germinated soy supplementation in the aqueous extract, which was still 12 times higher compared to a more efficient solvent extraction of celery using 75% ethanol, revealing that the conversion of apigetrin to apigenin was catalysed by the β-glucosidases present in germinated soy. This further demonstrates the feasibility of aglycone conversion using germinated soy as a source of β-glucosidases without the need for purification, making it a cost-effective approach. On top of that, the approach also contributes high concentrations of bioactive soy isoflavonoids such as genistein, daidzein, genistin and daidzin, which are reportedly beneficial for health [11,37].

## 4. Conclusions

In this study, the extraction conditions of bioactive aglycones from a celery extract supplemented with germinated soy were optimised by a response surface methodology. For subsequent enzymatic hydrolysis to enhance the apigenin content, increased production of its precursor apigetrin was firstly achieved through acidic extraction at optimal conditions involving water at pH 1 and 75 °C for 2 h. Subsequently, a central composite design was conducted to analyse the pH (3–11) and temperature (25–35 °C) effects on the apigenin, daidzein and genistein levels. The optimal extraction conditions were pH 7.02 and 29.99 °C and resulted in a 40-fold increase in apigenin. The novel and cost-effective application of germinated soy β-glucosidase for the conversion of aglycones in non-soy foods has been demonstrated, and the enhanced bioactivities of aglycones may suggest potential applications of similar formulations as functional food ingredients.

## Figures and Tables

**Table 1:** Experimental and predicted concentrations of apigenin, daidzein and genistein of the germinated soy-supplemented celery extract obtained by CCD. Experimental values are expressed as the average of triplicate determination from different experiments, while predicted values were calculated based on CCD evaluation.

Run	Parameters		Responses					
	X_1_ (pH)	X_2_ (T, °C)	Y_1_, Apigenin (ng/mL Extract)		Y_2_, Daidzein (µg/mL Extract)		Y_3_, Genistein (µg/mL Extract)	
			Exp	Pred	Exp	Pred	Exp	Pred
1	11.0	30.0	2.86	−3.76	2.38	3.91	9.66	19.32
2	7.0	30.0	38.87	35.97	6.37	7.83	22.97	30.96
3	4.2	33.5	26.82	27.16	5.32	5.12	13.77	15.14
4	7.0	35.0	33.29	30.79	7.37	8.13	26.97	30.38
5	7.0	25.0	37.47	33.48	6.86	7.58	27.56	30.28
6	7.0	30.0	34.12	35.97	8.32	7.83	32.74	30.96
7	3.0	30.0	19.54	19.67	2.50	2.45	7.22	3.68
8	9.8	26.5	6.34	12.50	7.04	5.76	33.62	26.13
9	4.2	26.5	28.11	29.50	5.05	4.88	13.78	15.63
10	9.8	33.5	5.92	11.03	7.61	6.30	34.75	26.77
11	7.0	30.0	31.95	35.97	7.72	7.83	30.49	30.96
12	7.0	30.0	38.94	35.97	8.92	7.83	37.65	30.96

**Table 2:** Polynomial equations and statistical parameters of the fitted models obtained for response variables.

Responses	Apigenin (Y_1_)	Daidzein (Y_2_)	Genistein (Y_3_)
Model Parameters			
Second-order polynomial equation	−154.82 + 20.93 X_1_ + 8.79 X_2_ + 0.02 X_1_X_2_ − 1.75 X_1_^2^ − 0.15 X_2_^2^	−6.92 + 4.02 X_1_ − 0.05 X_2_ + 0.01 X_1_X_2_ − 0.29 X_1_^2^ + 0.0009 X_2_^2^	−59.46 + 18.13 X_1_ + 1.33 X_2_ + 0.03 X_1_X_2_ − 1.22 X_1_^2^ − 0.03 X_2_^2^
R_2_	0.915	0.788	0.706
R_2_ (adjusted)	0.844	0.611	0.461
Lack of fit	0.161	0.304	0.263
*p*			
Model	0.004	0.048	0.115
X_1_ (pH)	0.055	0.115	0.209
X_2_ (T, °C)	0.424	0.984	0.932
X_1_X_2_	0.937	0.910	0.944
X_1_^2^	0.001	0.004	0.019
X_2_^2^	0.396	0.984	0.921

**Table 3:** Experimental and predicted response values at optimal conditions of pH 7.02 and 29.99 °C.

Responses	*p*	Predicted Value	Experimental Value
Apigenin (Y_1_)	0.467	35.97	38.97 ± 5.83
Daidzein (Y_2_)	0.201	7.83	6.90 ± 0.86
Genistein (Y_3_)	0.499	30.96	27.90 ± 6.49

**Table 4:** Key bioactive compounds in celery and soy obtained under optimal conditions in apigetrin-optimised celery extracts and germinated soy-supplemented celery extract.

Compound	Concentration		
	Celery Extract (75% ETOH)	Celery Extract (0% ETOH)	Germinated Soy-Supplemented Celery Extract
Apigenin (ng/mL)	3.17 ± 0.16	0.95 ± 0.05	38.97 ± 5.83
Apigetrin (ng/mL)	487.78 ± 11.58	183.88 ± 8.94	7.85 ± 1.25
Apiin (ng/mL)	222.89 ± 11.78	167.36 ± 13.58	6.83 ± 0.75
Genistein (µg/mL)	-	-	27.90 ± 6.49
Daidzein (µg/mL)	-	-	6.90 ± 0.86
Genistin (µg/mL)	-	-	3.34 ± 0.37
Daidzin (µg/mL)	-	-	20.18 ± 1.80

## Data Availability

Data are contained within the article or supplementary material.

## Supplementary Materials

The following are available online at www.mdpi.com/article/10.3390/foods10102505/s1, Table S1: MS parameters of celery and soy compounds.
