# Supplementary material for: Optimisation of an Aglycone-Enhanced Celery Extract with Germinated Soy Supplementation Using Response Surface Methodology
Source: Foods. 2021 Oct 19;10(10):2505. doi: 10.3390/foods10102505 (PMC8535585; doi:10.3390/foods10102505)
Supplement: Supplementary file 1 [file foods-10-02505-s001.zip › foods-1393834-supplementary.pdf]

Table S1. MS parameters of celery and soy compounds

Analyte	MW	tR (min)	Ionisation mode	Q1 (m/z)	Q3 (m/z)	DP (V)	CE (eV)	CXP (V)	Linear equation	Linearity range (ng/mL)	R^2	LOD (ng/mL)	LOQ (ng/mL)
Daidzin	416	3.96	+	417	255	60	30	14	y = 49734x + 6568	2.5 - 50	1.000	1.17	3.53
Genistin	432	4.22	+	433	271	50	23	15	y = 235068x - 13821	0.5 - 10	1.000	0.32	0.96
Apiin	564	4.56	+	565	271	40	32	15	y = 73452x - 86991	2.5 - 100	1.000	1.77	5.36
Apigetrin	432	4.65	+	433	271	50	25	15	y = 112017x - 35644	2.5 - 50	1.000	1.52	4.62
Daidzein	254	5.1	+	255	199	100	34	10	y = 179372x + 89405	0.5 - 25	0.997	0.21	0.63
Genistein	270	5.56	-	269	133	140	38	7	y = 17718x - 14517	2.5 - 50	1.000	1.57	4.75
Apigenin	270	5.93	-	269	117	100	45	6	y = 46649x - 32157	1 - 50	0.998	0.43	1.28
